# Case report: Episodic psychosis caused by a novel *SCP2* splicing mutation

**DOI:** 10.3389/fneur.2023.1270793

**Published:** 2023-10-12

**Authors:** Haiyan Tang, Yingying Luo, Zhenchu Tang, Jianguang Tang, Jia Fang

**Affiliations:** ^1^The Second Xiangya Hospital, and Center for Medical Genetics and Hunan Key Laboratory of Medical Genetics, Department of Medical Genetics, School of Life Sciences, Central South University, Changsha, Hunan, China; ^2^Department of Neurology, The Second Xiangya Hospital of Central South University, Changsha, China

**Keywords:** episodic psychosis, peroxisomal beta-oxidation, SCPx deficiency, SCP2 mutation, splicing mutation

## Abstract

SCPx deficiency is a rare disorder of peroxisomal beta-oxidation dysfunction, and it has only been documented in two patients thus far. In the previously reported patients, both patients were primarily presented with slowly progressive dystonia or ataxia, and they both displayed symmetrical lesions in the thalamus and brainstem on magnetic resonance imaging. This study presents the third patient exhibiting a similar neuroimaging abnormality but a notably different clinical phenotype characterized by episodic psychosis. Through whole-exome sequencing, we identified a homozygous splicing mutation in *SCP2* (c.674 + 1G > C), and further RNA sequencing revealed exon 8 skipping in the mature transcripts of *SCP2*. This study significantly expands our understanding of the genotypic and phenotypic spectrum associated with *SCP2-*related metabolic encephalopathy.

## Introduction

*SCP2* gene is located on chromosome 1p32 and encodes two distinct proteins, sterol carrier protein X (SCPx) and sterol carrier protein 2 (SCP2), *via* separate promoters (1). SCPx functions as a peroxisomal enzyme with thiolase activity involved in peroxisomal beta-oxidation. Its primary role is in the degradation of very long-chain fatty acids (VLCFA) and branched-chain fatty acids such as phytanic and pristanic acid. In the current study, we reported the case of a patient who presented with episodic psychosis, which was determined to be caused by a novel *SCP2* splicing mutation.

## Case description

**Figure 1:**
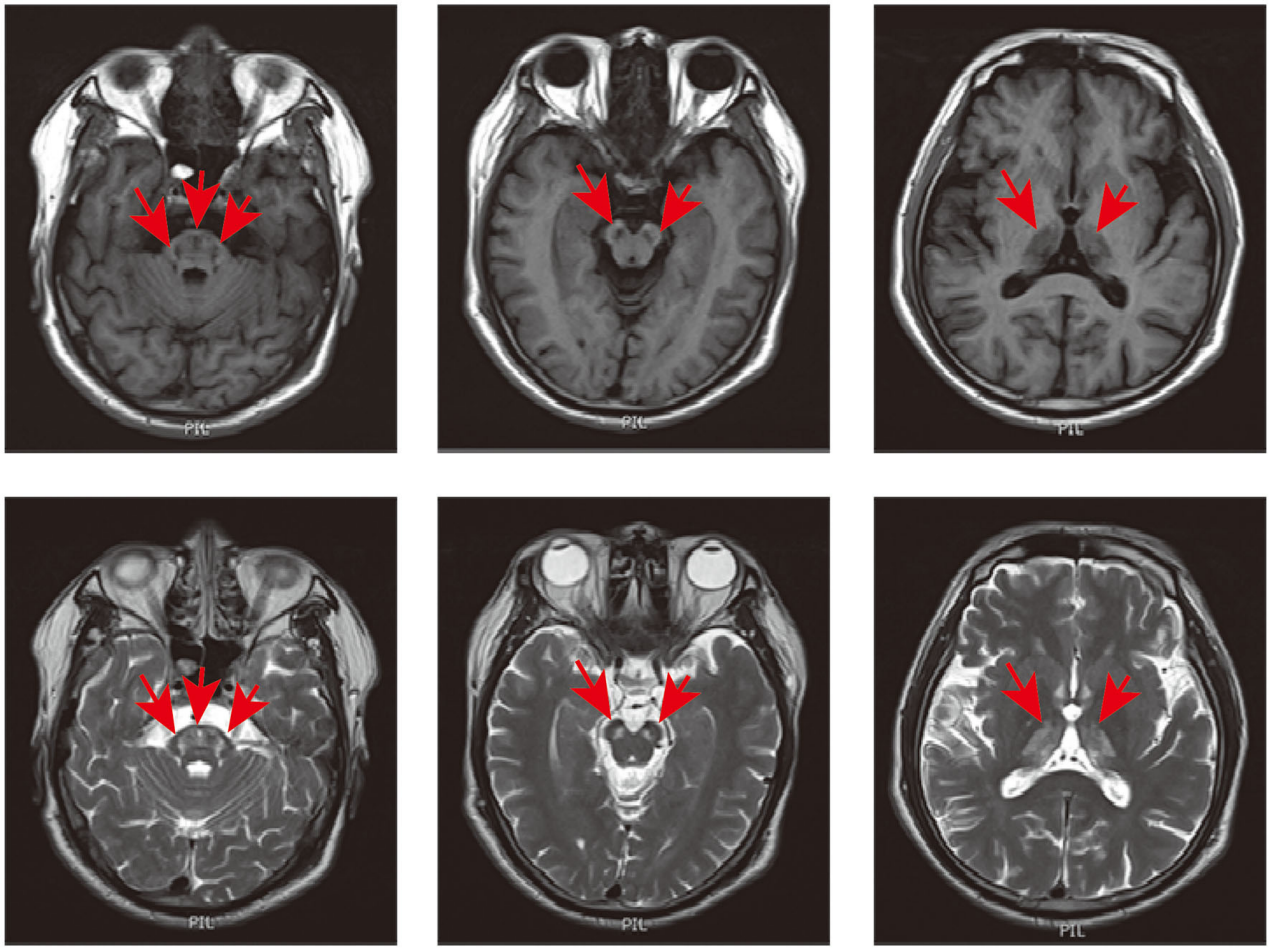
Cranial MRI of the patient. Axial T1 and T2-weighted imaging showed hypointense and hyperintense signals in the thalamus, mesocephalon, and pons (indicated in red arrow).

Ethics approval was obtained from the Second Xiangya Hospital, Central South University (Changsha, China). Written informed consent was obtained from the patient and his family members for their enrollment. A 48-year-old male was admitted to our hospital thrice between 2012 and 2017 due to episodic psychosis. During each attack, he experienced delusions, irritability, aggressive behavior, bursts of uncontrollable laughter, crying, and talking to himself. Each episode was triggered by an acute upper respiratory tract infection. He was the third child of healthy, non-consanguineous parents with no family history of relevant conditions. The patient had experienced stuttering and tremors in his lips and hands for as long as his son could remember. Physical signs were the same during each episode. Examination revealed confusion, restlessness, and disorientation to time, person, and place, with normal limb muscle strength, brisk deep tendon reflexes, positive bilateral palmomental reflex, sucking reflex, and Babinski's reflex. On each occasion, cranial magnetic resonance imaging (MRI) revealed symmetrical T2 hyperintense signals in the thalamus, mesocephalon, and pons without gadolinium enhancement (see Figure 1). The cerebrospinal fluid (CSF) showed no abnormalities. No antibodies were detected for autoimmune encephalitis, paraneoplastic neurological syndromes, and rheumatologic disorders. Ceruloplasmin, lactic acid, free carnitine, acylcarnitines, amino acids in the blood, and organic acids in the urine were all within normal ranges. Nerve conduction studies and needle electromyography showed no abnormalities. The patient was previously diagnosed with viral encephalitis, cerebral infarction, or mitochondrial encephalopathy. He received appropriate treatments, such as antiviral and antiplatelet therapy, during each hospitalization. Psychiatric symptoms gradually improved after 3–6 months of treatment with antipsychotic drugs such as olanzapine or quetiapine. Between episodes, the patient was able to care for himself and engage in communication with others independently.

In January 2018, the patient was readmitted to the hospital for continuous generalized tonic-clonic seizures (GTCS) that lasted 18 h without recovery between seizures. The GTCS occurred every 10 min, with each seizure lasting 1–2 min. Upon admission, he presented with confusion. The physical examination was limited due to poor cooperation. Pristanic acid concentration was slightly elevated at 0.62 μmol/L, just above the control range (<0.60 μmol/L), while VLCFA and phytanic acid were within the standard limit. Cranial MRI revealed abnormalities consistent with prior findings.

**Figure 2:**
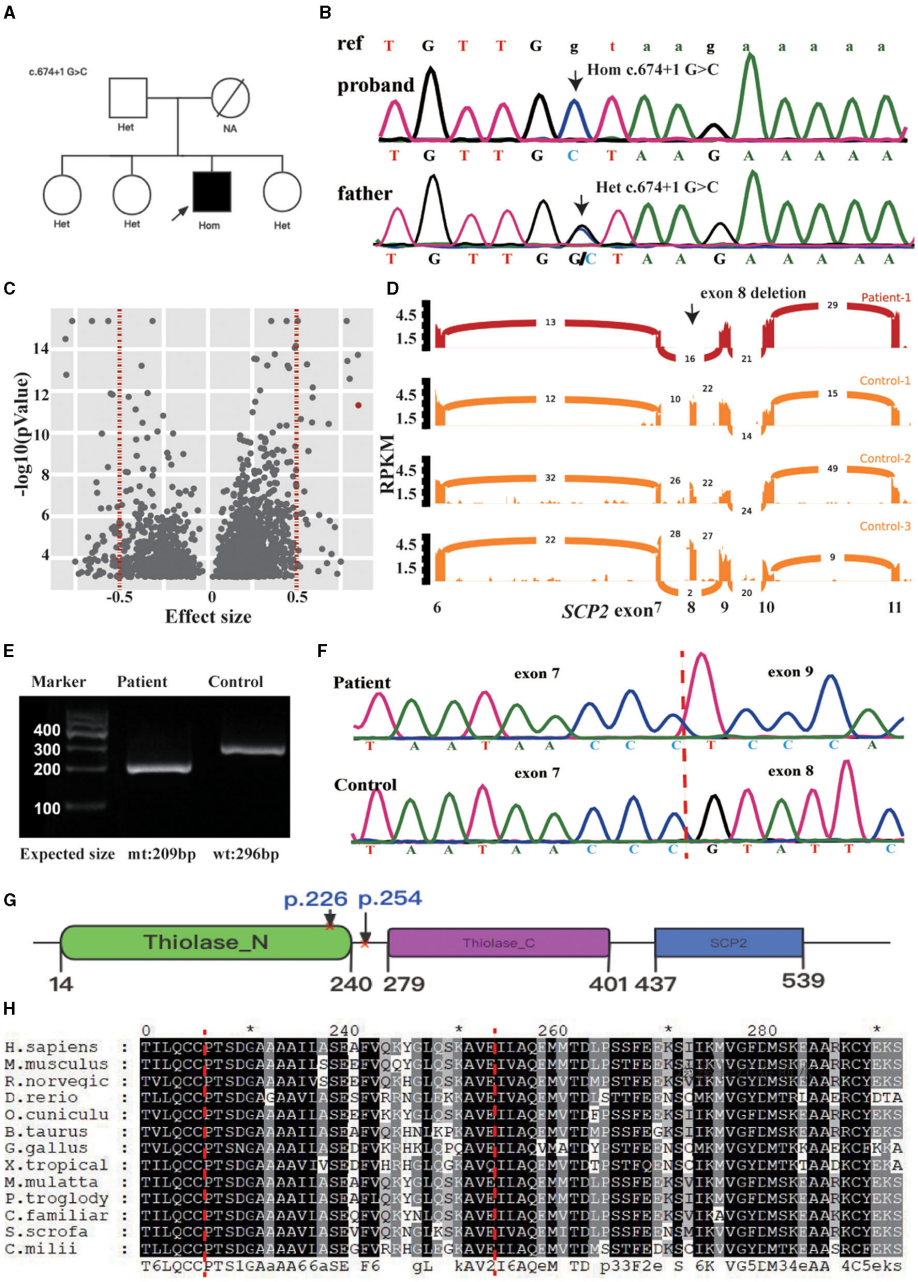
**(A)** Pedigree chart of this family. **(B)** Validation of *SCP2* c.674 + 1 G>C by Sanger sequence in the patient and his father. The ref indicates the reference sequence, with capital letters and lowercase, representing the nucleotide in the exon and intron regions. **(C)** Outlier-level significance [-log10(p), y-axis] vs. effect size (junction count/total junction count-mean (total junctions count/total count of junctions in the cluster), x-axis) for the patient. There are 2030s outliers with an adjusted *p* < 0.05 in 1442 genes (red dots indicating two clusters in *SCP2*, vertical dotted lines indicating the effect size cutoff). **(D)** Sashimi plot of the eighth exon-skipping event (indicated in black arrow) in RNA-seq samples of the *SCP2*-affected (red) and three representative *SCP2*-unaffected (orange) individuals. The RNA-seq read coverage is given as the log10 RPKM-value (Reads Per Kilobase of transcript per Million mapped reads, y-axis), and the number of splits reads spanning an intron is indicated on the exon-connecting line. **(E)** The skipping event is observable in the RT-PCR product from patient blood. Mt: mtant type, wt: wide type. **(F)** Exon 8 skipping is validated by the Sanger sequencing of RT-PCR product. **(G)** schematic representation of the SCPx and the affected domain of the index patient, surrounding the amino acids region coding by the eighth exon (p. 225–254, indicated between the red cross). **(H)** Protein sequence alignment of *SCP2* orthologs, showing the region surrounding the p. 225–254 is relatively conserved (indicated between the red dot lines). ^*^Indicates the termination codon.

Whole-exome sequencing of the proband revealed a homozygous splicing mutation, c.674 + 1G > C, in *SCP2* (NM_002979.5) in chr1:53442442 (hg19). Sanger sequencing confirmed the candidate mutation in the proband and his family members. The proband's father and sisters were heterozygotes at this position (see Figures 2A, B). RNA sequencing identified two outlier junctions in the *SCP2* cluster, providing support for the skipping of the eighth exon (*p* = 4.81E-12, FDR = 0, and *p* = 1.31E-4, FDR = 0.008) (see Figures 2C, D). At the mRNA level, cDNA analysis validated the aberrant splicing pattern between the sixth and ninth exons in the patient's sample through the agarose gel electrophoresis of reverse transcription polymerase chain reaction amplicons (RT-PCR) product (see Figure 2E). Sanger sequencing of the RT-PCR product confirmed the exon 8 skipping event in the patient (see Figure 2F). The eighth exon has a length of 87 base pairs. This mutation results in the loss of 29 amino acid residues in the patients' SCPx protein. The allele frequency is approximately 0.00001992 in the gnomAD database. In the case of SCP2, loss of function is a recognized disease mechanism. The variant c.674 + 1G > C in *SCP2* was classified as “pathogenic” based on the ACMG guidelines.

The patient's seizures were effectively halted with diazepam, and the psychiatric symptoms gradually improved. Subsequently, the patient was discharged while being placed on a phytanic acid-restricted diet.

## Discussion

SCPx deficiency, a rare autosomal recessive monogenic metabolic encephalopathy, has been reported in only two patients. One patient presented with leukoencephalopathy with clinical features such as slowly progressive dystonia, stutter, and motor neuropathy (2). The other patient developed hand clumsiness in his 30s, followed by gait disturbance and deafness (3). The index patient displayed episodic psychobehavioral disturbances, stutter, tremors, and epilepsy. MRI findings were consistent among all three patients, revealing symmetrical thalamus and brainstem lesions without gadolinium enhancement. As summarized in Table 1, these shared clinical features in SCPx deficiency include stutter, tremors, and symmetrical lesions in the thalamus and brainstem without gadolinium enhancement (see Table 1). Importantly, our patient first presented with episodic psychosis and epilepsy in SCPx deficiency patients highlighting the necessity for clinicians to consider the *SCP2* variant during the etiological examination of individuals presenting with episodic psychosis and epilepsy.

**Table 1:** Clinical phenotypes and laboratory investigation results of patients with *SCP2* mutations.

**Patient ID**	**Patient 1^2^**	**Patient 2^3^**	**Index Patient**
Gender	Male	Male	Male
Age at disease onset	7 years	30 years	Young, not clear
Clinical neurology symptoms	torticollis, tremor, nystagmus, hyposmia	hand clumsiness, gait disturbance, deafness	episodic psychobehavioral disturbances, stutter, tremor, epilepsy
Neurological examination	hyposmia, pathological saccadic eye movements, brisk deep-tendon reflexes of the upper extremities but diminished reflexes of the lower extremities, plantar sole responses, and a reduced vibration sense of 4/8 at the lateral malleoli	slow ocular saccades, muscle tone was increased with brisk deep tendon reflexes throughout, proprioception was impaired, mild dysmetria, dysdiadochokinesis, and a wide-based gait	Inaccurate orientation, slightly slow response, talking nonsense, and involuntary shaking of lips and upper limbs, positive right ankle clonus, double metacarpophalangeal reflex and sucking reflex positive, bilateral Babinski sign and allelic sign positive.
Cranial MRI results	bilateral hyperintense T2 signals in the thalamus, butterfly-like lesions in the pons, and lesions in the occipital region, without gadolinium enhancement	abnormal T2 signal, in the pons and thalamus	bilateral hyperintense T2 signals in the thalamus, and butterfly-like lesions in the pons
Electrophysiology	a predominantly motor and slight sensory neuropathy, with conduction blocks in the tibial nerves, reduced motor action potentials in the left peroneal nerve, and reduced amplitude of the left sural nerve	No evidence of polyneuropathy	No evidence of polyneuropathy
Pristanic acid	39.8 μmol/ L, control range 0–3.1	34.1 μmol/L, normal range 0–1.5	0.96 μmol/L, control range < 0.6
Phytanic acid	10.1 μmol/L, control range 0–9	13.8 μmol/L, normal < 11.5	0.74 μmol/L, control range < 5.7
Genetics	c.545_546insA(hom);	c.121G>T &c.349C>T(comhet);	c.674 + 1g>c(hom);
	p.I184fs^*^7	p.Glu41^*^&p.Gln117^*^	27 amino acids deleted

Patients 1 and 2 described in the references (2, 3), and index patient described in the study. (^*^ Indicates the termination codon).

Patients in the literature exhibited elevated pristanic acid levels, whereas the index patient showed a mild increase. Mutations in *HSD17B4*, which resulted in less structural damage to the D-bifunctional protein, were associated with a milder clinical and biochemical presentation (4). The D-bifunctional protein is situated upstream of SCPx in peroxisomal beta-oxidation. In our patient, the deletion of amino acid residues due to exon skipping occurs within a relatively conserved region of the protein, and this deletion region intersects with the thiolase_N domain (see Figures 2G, H). The function of the thiolase may be impaired but remains partially functional.

Intriguingly, episodic psychosis in the index patient typically follows infections, indicating that “crises” in SCPx deficiency may be provoked by factors such as infections or extended fasting, similar to certain metabolic encephalopathies, such as isolated methylmalonic acidemia (5).

## Data availability statement

The original contributions presented in the study are included in the article/supplementary material, further inquiries can be directed to the corresponding author.

## Ethics statement

The studies involving humans were approved by the Second Xiangya Hospital, Central South University (Changsha, China). The studies were conducted in accordance with the local legislation and institutional requirements. The participants provided their written informed consent to participate in this study. Written informed consent was obtained from the individual(s) for the publication of any potentially identifiable images or data included in this article.

## Author contributions

HT: Formal analysis, Writing—original draft. YL: Software, Writing—review and editing. ZT: Data curation, Writing—review and editing. JT: Investigation, Writing—review and editing. JF: Investigation, Supervision, Writing—original draft.
